# Safety and Efficacy of Ginkgo-Damole and Nitroglycerin or Sodium Nitroprusside on Hypertensive Cerebropathies: A Meta-Analysis

**DOI:** 10.1155/2020/5713796

**Published:** 2020-09-04

**Authors:** Li Peng, Wei-kun Zhao, Tong-tong Xu, Qi Wu, Pan Lu, Pan-pan Zhu, Xiao-ming Zheng

**Affiliations:** ^1^Department of Health Care Ward, Affiliated Hospital of Guilin Medical University, Guilin, Guangxi Zhuang Autonomous Region 541001, China; ^2^Department of Physiology, Xuzhou Medical University, Xuzhou 221009, China

## Abstract

**Objective:**

To systematically evaluate the safety and efficacy of ginko-damole combined with nitroglycerin or unitary sodium nitroprusside on hypertensive cerebropathy.

**Methods:**

Four Chinese databases (VIP, CBM, Wanfang database, and CNKI database) and three English databases (Cochrane, PubMed, and EMBASE) were used to screen randomised controlled trials (RCTs) on treatments of hypertensive cerebropathy using both ginko-damole and nitroglycerin or unitary sodium nitroprusside. Outcomes included clinical effect, blood pressure after treatment, and adverse effects. These indicators were then analysed statistically using the RevMan 5.3 and Stata 12.0 software.

**Results:**

Altogether, 16 RCTs including 1507 patients with hypertensive cerebropathy were included in the present meta-analysis, of which, 755 patients treated with combined ginko-damole and nitroglycerin were included in the observation group and 752 patients treated with sodium nitroprusside were included in the control group. The curative effect of the observation group was significantly better than that of the control group (RR: 1.115 [1.077, 1.155], *p* < 0.05). DBPs of the observation and control groups were both lower after treatment, and no significant difference was observed between the observation and control groups (MD: −1.072 [−2.578, 0.434], *p* > 0.05). SBPs in the observation group were significantly lower than those in the control group (MD: −2.842 [−5.222, −0.462], *p* < 0.05). The probability of adverse response in both groups did not differ significantly (RR: 0.752 [0.412, 1.374], *p* > 0.05).

**Conclusion:**

Compared with sodium nitroprusside, the combined ginkgo-damole and nitroglycerin could better control blood pressure in patients with hypertensive cerebropathy and showed enhanced clinical effects and improved safety. However, due to poor quality of the included studies, results of the present meta-analysis should be confirmed by more stringent RCTs.

## 1. Introduction

Owing to the pathogenesis of primary or secondary hypertension or under the effect of some stressors, the illness may worsen because of a dramatic increase in blood pressure, leading to hypertensive emergency, whereby the heart, brain, kidney, and other target organs may be affected. Hypertensive emergency may occur in 1%-2% of patients with hypertension [1]. Neurovascular emergencies are the main reason for sudden death of patients with hypertensive crisis [2]. Hypertensive encephalopathy is characterized by neurological symptoms such as headache, mesis, and disturbance of consciousness. Hypertensive encephalopathy is an emergency situation wherein blood pressure rises above the regulatory threshold (central arterial pressure >140 mmHg), cerebral perfusion pressure elevates beyond the autoregulation range, leading to fibrinoid necrosis of arterial tissue cells and vascular damage, leading to the destruction of the and blood-brain barrier, eventually causing cerebral ischaemia, encephaloedema, increased intracranial pressure, and cerebral herniation [3, 4]. When mean artery pressure ≥180 mmHg, body's autoregulation mechanism responds; the previously contracted blood vessels due to increased blood pressure will be passively expanded, leading to cerebral vascular over reperfusion and vasogenic cerebral oedema. When vasogenic cerebral oedemaedoema occurs, timely, effective, and safe blood pressure reduction can prevent its spread, which may benefit patients' prognosis. In contrast, late or improper treatment may lead to persistent perfusion and further enhanced vascular wall permeability, which may damage vascular endothelial cells and cause degeneration, cytotoxic cerebral oedemaedoema, and even cerebral infarction or cerebral haemorrhage.

Ginkgo is a perennial woody plant, whose leaves and seeds are used in Chinese medicine. The extract of *Ginkgo biloba* L. (Ginkgoaceae) is recorded in the pharmacopeia and functions by dilating coronary and cerebrovascular vessels, thus improving cerebral ischaemia and memory function and is frequently used as an ancillary medicine for the treatment of cerebral diseases [5, 6]. Dipyridamole can inhibit the platelet aggregation and dilate coronary vessels. Ginkgo-damole is a compound preparation derived from the active ingredient of *Ginkgo biloba* L., ginkgo flavonol, and dipyridamole. A 5 ml ginkgo-damole injection contains 4.5–5.5 mg of ginkgo flavonol and 1.8–2.2 mg of dipyridamole. Ginkgo-damole has been widely used for the ancillary treatment of acute cerebral infarction, angina pectoris, and thromboembolism-related diseases [7–10]. Sodium nitroprusside can relax smooth muscles, expand veins and arteries, and reduce the front and rear loads to decrease the blood pressure by promoting NO release from the smooth muscle cells. However, sodium nitroprusside can produce cyanide through red blood cell metabolism, and long-term usage or large dosing may lead to thiocyanic acid poisoning and cardiac toxicity. Similarly, high doses of nitroglycerin also have a powerful effect on expanding veins and selectively expand the coronary and main artery, thereby reducing adverse reactions resulting from sodium nitroprusside administration including nausea, vomiting, and muscle vibration and cardiac toxicity due to excessive sodium nitroprusside dosing. Unitary administration of nitroglycerin usually leads to headaches, face flushes, and tachycardia [11]. Dipyridamole can inhibit phosphodiesterase-5 (PDE-5) activity, block cyclic guanosine monophosphate (cGMP) degradation, promote vessel dilation, and activate parasympathetic nerves. The combined use of dipyridamole and nitroglycerin can probably lower the heart rate. Studies in animal models suggested that combined administration of dipyridamole and ginkgo-damole could promote NO release from the endothelial cells, thereby decreasing arterial blood pressure [12]. Another study in dogs reported that both 3 mg/kg and 9 mg/kg of ginkgo-damole could lower the heart rate and decrease the blood pressure [13].

The present study aimed to collect RCTs conducted worldwide on treatments of hypertensive cerebropathy using ginkgo-damole and nitroglycerin or sodium nitroprusside to systematically assess its safety and curative effects and provide evidence for its clinical use in hypertensive cerebropathy.

## 2. Data and Methods

### 2.1. Literature Search

The literature was searched in the following seven electronic databases Cochrane, PubMed, Excerpta Medica Database (Embase), China National Knowledge Infrastructure (CNKI), Value In Pape (VIP), Wanfang database, and China Biology Medicine (CBM). Registered clinical trials were searched in the website of the National Institutes of Health of America (http://clinicaltrials.gov/), China Clinical Trials Registration Centre (http://www.chictr.org/), and International Clinical Trials Registration Centre. Papers were included dated from the construction time of each database until February 22, 2020. The following keywords were used in single or combination treatment: ginkgo-damole, Ginkgo Damo, Yinxing Damo, ginkgo leaf extract and dipyridamole, effect of *Ginkgo biloba* extract and dipyridamole, nitroglycerin, the extract of *Ginkgo biloba* L., leaf extract of *Ginkgo biloba*, leaf extract of ginkgo leaf, *Ginkgo biloba*, blood pressure, hypertension, hypertensive cerebropathy, hypertensive encephalopathy, hypertensive emergency, hypertensive crisis, malignant hypertension, cardiovascular vessel, cerebrovascular vessel, cardiocerebro vascular vessel, clinical trials, randomised controlled trials, dipyridamole, blood circulation, blood circulation improvement, and stasis removal. The literature cited by included publications was also searched.

### 2.2. Inclusion Criteria of Literatures

#### 2.2.1. Type of Studies

RCTs used for the treatment of hypertensive cerebropathy using combined ginkgo-damole and nitroglycerin published before February 22, 2020 were included in the present meta-analysis.

#### 2.2.2. Study Objects

According to the diagnostic criteria of hypertensive encephalopathy [3], all patients were consistent with the diagnostic standard for hypertensive cerebropathy.

#### 2.2.3. Preventative Measures

The observation group was treated with combined ginkgo-damole and nitroglycerin, while the control group was treated with sodium nitroprusside.

#### 2.2.4. Outcome Indicators

At least one of the following items, total effect, level of blood pressure, and incidence of adverse response, was included.

#### 2.2.5. Exclusion Criteria of Literatures

Literature was excluded by the following criteria: (1) undiagnosed cases of hypertensive cerebropathy, (2) duplicated publications, (3) inaccessible full-text, (4) literature with missing information on cases and treatments, and (5) animal tests.

### 2.3. Data Extraction

Literature search, study selection, and data extraction were completed by two research fellows. The extracted information was recorded in a table according to predefined standard and then subjected to cross-check. All disagreements were resolved by involving a third party for agreement.

### 2.4. Literature Quality Assessment

Quality of the methodology was assessed using the Cochrane standard [14]. This assessment included the following methods, sequence generation, allocation concealment, blinding, incomplete outcome data, selective outcome reporting, and other sources of bias. The risk of every method was assessed, and the risk assessment table was prepared using the Revman 5.3 software.

### 2.5. Statistic Analysis

Data were analysed using RevMan5.3 and Stata12.0. Binary outcome data are expressed as risk ratio (RR). Consecutive variables are expressed as mean value ± standard deviation (SD) and 95% confidence interval. The chi-squared test and *I*^2^ test were used to test heterogeneity. *p* > 0.1 and *I*^2^ < 50% represented the homogeneity of the combined treatment, and a fixed-effect model was used for the meta-analysis. *p* ≤ 0.1 and *I*^2^ ≥ 50% represented heterogeneity of the combined treatment, and a random-effect model was used for meta-analysis. Publication bias was evaluated using Begg's test, and *p* > 0.05 represented the inexistence of publication bias. If the number of included RCTs was sufficient, a sensitivity test was used to test the stability of the results of the meta-analysis.

## 3. Results

### 3.1. Results of Literature Search

**Figure 1:**
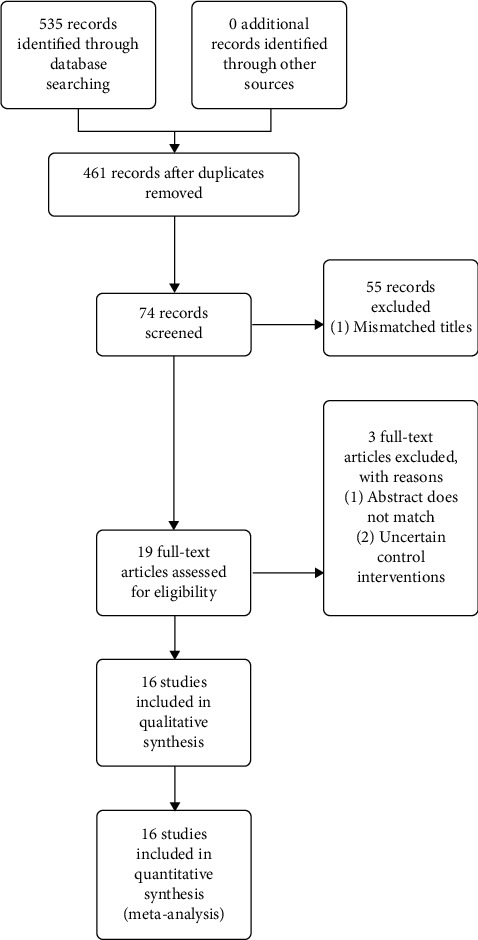
Flowchart showing the process of study selection.

According to our search strategy, a total of 535 papers were found, of which 461 papers were removed after screening. Fifty-five papers were excluded based on titles, and two more based on abstract reading. Finally, one paper was excluded after full reading because of unclear preventative measure (Figure 1).

### 3.2. Basic Traits of Included Studies

The basic information included author name, research title, year of publication, preventative measure, number of patients in the experimental and control groups, and outcome indicators. Of the 16 RCTs, 755 patients were included in the observation group and 752 patients in the control group. Patients in the control and observation groups were offered sedatives, oxygen, anodyne, and intracerebral pressure control. Meanwhile, the control group received sodium nitroprusside to reduce patient's blood pressure by 25%. When patient's blood pressure was reduced to 160/100 mmHg, the patient received oral antihypertensives. In contrast, patients in the observation group received nitroglycerin. When patient's blood pressure was reduced to 160/100 mmHg, ginkgo-damole was transfused at 20 mL/250 mL (Table 1).

### 3.3. Bias Risk Assessment of Included Literatures

**Figure 2:**
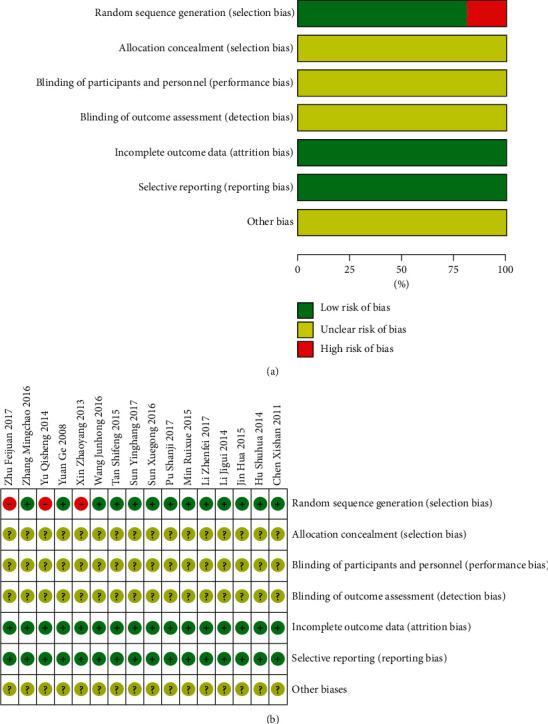
Bias risk analysis of the included studies. (a) Methodological quality assessment of all included studies. (b) Summary of methodological quality assessment of each included study. +: L (low risk of bias); ?: U (unclear risk of bias); −: H (high risk of bias).

Thirteen out of the 16 RCTs mentioned randomised control, and three of those mentioned the methods of randomisation. However, none of these 16 RCTs mentioned allocation concealment or a blind method, nor they mentioned other sources of bias (Figures 2(a) and 2(b)).

### 3.4. Clinical Effect

**Figure 3:**
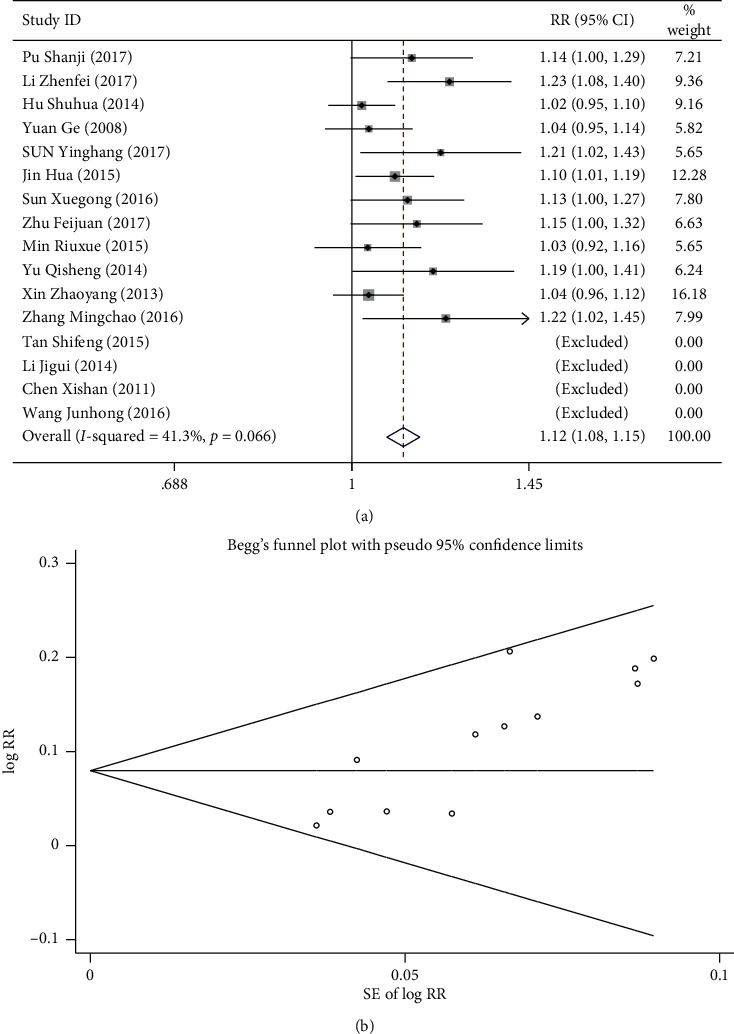
Comparison of clinical efficacy between the combined ginkgo-damole and nitroglycerin treatment group and control group. (a) Forest plots comparing clinical efficacy between the groups. (b) Funnel plot showing publication bias of clinical efficacy between the groups using Begg's rank correlation test.

A total of 16 RCTs [15–30] compared the clinical effect of the observation and control groups. Three grades, as recommended by the Guiding Principles for Clinical Research of Cardiovascular System Drugs in the “Guiding Principles for Drug Clinical Research” of the Ministry of Health, were used to evaluate the therapeutic effects of combined ginkgo-damole and nitroglycerin or sodium nitroprusside for hypertensive cerebropathy in 12 trials [15, 16, 18–21, 23, 25, 26, 28–30]. Significant effect means disappearance of clinical symptoms and a reduction in diastolic and a systolic pressure of >20 mmHg. Effective means clear improvement in clinical symptoms and a reduction in diastolic and systolic pressure of 10–20 mmHg. In case of simple systolic hypertension, a decrease in systolic blood pressure ≥30 mmHg is significant and effective. Noneffect means no significant improvement in clinical symptoms, even deterioration while blood pressure keeps increasing. There are four articles [17, 22, 24, 27] divided into four levels according to improvement of blood pressure and clinical symptoms. Recovery is defined as a complete disappearance of clinical symptoms and normalization of the blood pressure. A significant effect is an improvement in clinical symptoms to a certain extent. Effective means almost disappearance of clinical symptoms. Noneffect means no change in clinical symptoms and even deterioration. To guarantee an analysis, we transformed this information into binary data. The recovery and significant effects were both considered effective. *I*^2^ = 41.3 and *p* < 0.1 represent homogeneity, and a fixed-effect model was employed in turn. Results of meta-analysis showed that the curative effect of the observation group was significantly higher than that of the control (RR: 1.115[1.077, 1.155], *p* < 0.05) (Figure 3(a)). However, the results of Begg's test suggested publication bias (*p* < 0.05; Figure 3(b)).

### 3.5. Blood Pressure

**Figure 4:**
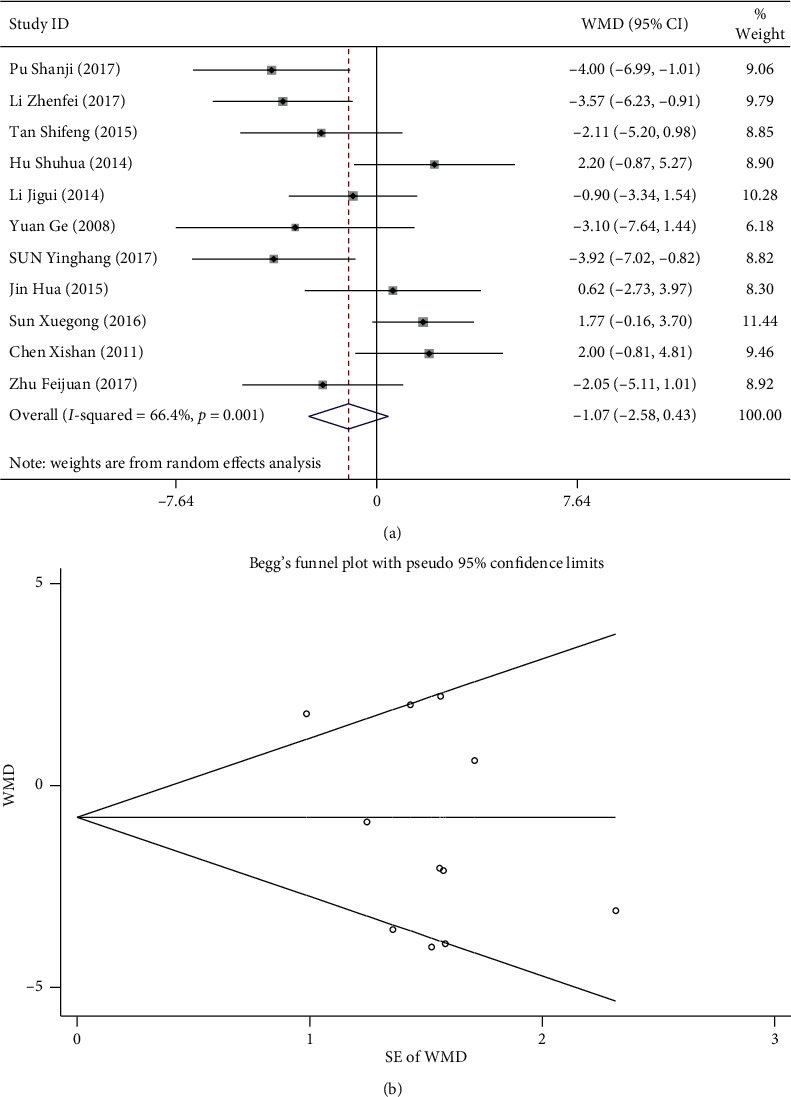
Comparison of DBP between the combined ginkgo-damole and nitroglycerin treatment group and control group. (a) Forest plots comparing DBP between the groups. (b) Funnel plot showing publication bias of DBP between the groups using Begg's rank correlation test.

**Figure 5:**
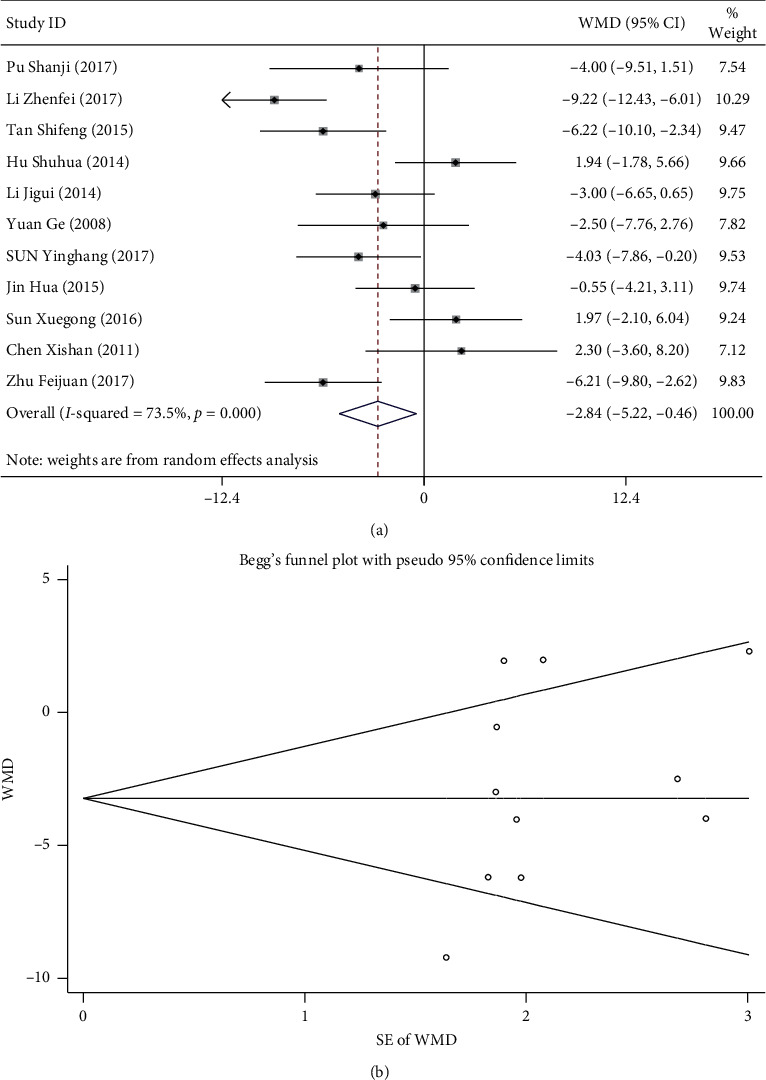
Comparison of SBP between the ginkgo-damole and nitroglycerin treatment group and control group. (a) Forest plots comparing SBP between the groups. (b) Funnel plot showing publication bias of SBP between the groups using Begg's rank correlation test.

A total of 11 studies compared the blood pressure of the observation and control groups [15, 16, 18, 23–30]. For DBP, there was *I*^2^ = 66.4% and *p* < 0.1, suggesting homogeneity, and the fixed-effect model was adopted. The results of meta-analysis showed that DBP in both groups was reduced after treatment. No significant difference was observed between the observation and control group (MD: −1.072 [−2.578, 0.434], *p* > 0.05) Figure 4(a). Begg's test did show publication bias (*p* > 0.05; Figure 4(b)). For SBP, there was *I*^2^ = 73.5 and *p* < 0.1, suggesting heterogeneity, and the random-effect model was adopted. Results of meta-analysis showed that SBP of the observation group was significantly lower than that of the control group (MD: −2.842 [−5.222, −0.462], *p* < 0.05) (Figure 5(a)). Results of Begg's test did not suggest any publication bias (*p* > 0.05; Figure 5(b)).

### 3.6. Safety

**Figure 6:**
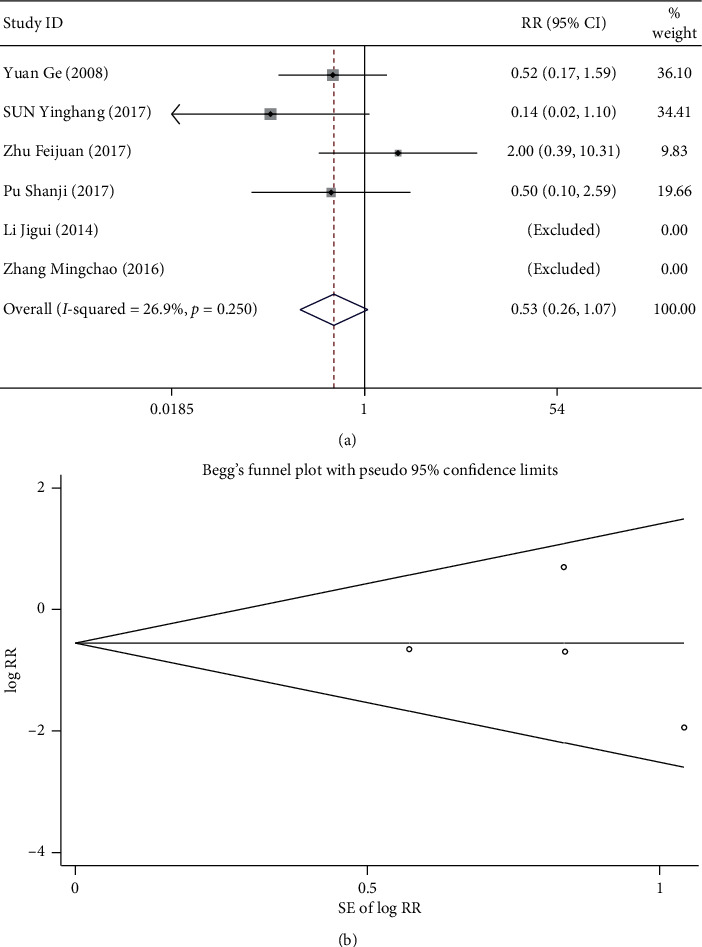
Comparison of safety between the combined ginkgo-damole and nitroglycerin treatment and control groups. (a) Forest plots for comparisons of safety between the groups. (b) Funnel plot for the publication bias of safety between the groups using Begg's rank correlation test.

A total of seven studies compared safety in the observation and control group [15, 18, 21, 24, 26, 28, 29]. The adverse response of patients included in this study includes aggravated headache, dizzy, face flush, anterior cardiac area tension, nausea, vertigo, and orthostatic hypotension. The *I*^2^ = 47.5% and *p* > 0.1 suggest homogeneity, and the fixed-effect model was used. Results of meta-analysis suggested a similar probability of adverse response in both groups. (RR: 0.752 [0.412, 1.374], *p* > 0.05) (Figure 6(a)). The results of Begg's test did not suggest a publication bias (*p* > 0.05; Figure 6(b)).

## 4. Discussion

In this study, a meta-analysis was used to evaluate the systolic pressure, diastolic pressure, clinical efficiency, and safety when patients with hypertensive encephalopathy were treated either using unitary sodium nitroprusside or a combination of ginkgo-damole and nitroglycerin.

Results from five studies [23, 25, 28, 29] showed that the combined treatment of ginkgo-damole and nitroglycerin had a positive effect in reducing BP as compared with that with sodium nitroprusside. However, no significant difference was observed in the other six studies [15, 16, 18, 24, 26, 27, 30] when a combination of ginkgo-damole and nitroglycerin was administrated to reduce SBP and DBP as compared with the effect of unitary sodium nitroprusside. In the present meta-analysis, we compared the blood pressure-reducing effect of the combined ginkgodamole and nitroglycerin or sodium nitroprusside. The results showed that combined administration of ginkgo-damole and nitroglycerin led to a significantly higher reduction of systolic pressure in patients with hypertensive cerebropathy than sodium nitroprusside. Combined administration of ginkgo-damole and nitroglycerin had a similar effect in reducing diastolic pressure in comparison with control. Results of Begg's test did suggest publication bias. Therefore, combined ginkgo-damole and nitroglycerin could be administrated to reduce the blood pressure of patients with hypertensive cerebropathy.

A total of 11 studies [16, 17, 19, 21, 22, 24–26, 28–30] showed an enhanced treatment effect of the combined dosage of ginkgo-damole and nitroglycerin as compared with that of unitary sodium nitroprusside for hypertensive encephalopathy. In another 5 studies [15, 18, 20, 23, 27], the combination of ginkgo-damole and nitroglycerin had the same treatment effect as that of sodium nitroprusside for hypertensive encephalopathy. However, these 5 studies suggest that the combination of ginkgo-damole and nitroglycerin could better help completely resolve the clinical symptoms of patients with hypertensive encephalopathy and restabilise their blood pressure. Hypertensive cerebropathy is characterized by headache, consciousness change, and spasm. The results of meta-analysis showed that combined administration of ginkgo-damole and nitroglycerin significantly improved clinical symptoms. However, the results of Begg's test suggested some publication bias, which may have affected the reliability of results.

A total of 7 studies assessed the safety of combined ginkgo-damole and nitroglycerin. Nitroglycerin showed that the combined treatment for hypertensive cerebropathy nitroglycerin had a similar probability of adverse responses as sodium nitroprusside. All of these RCTs included did not show severe adverse responses. And, the adverse responses could be alleviated or disappeared after a reduction in the instillation rate or drug withdrawal. Meanwhile, results of Begg's test did not suggest publication bias. This suggests that ginkgo-damole is probably safe for the treatment of hypertensive cerebropathia.

Our meta-analysis on the efficiency and safety of sodium nitroprusside and the combination of ginkgo-damole and nitroglycerin for hypertensive encephalopathy included a total of 17 RCTs and 1507 participants. The results showed that the combination of ginkgo-damole and nitroglycerin had a better efficacy than unitary sodium nitroprusside, specifically reflected in the following: (1) systolic pressure was significantly reduced; (2) nervous system symptoms including headache, vomiting, and altered consciousness were alleviated; (3) adverse events such as intensified headache, giddiness, facial hot flush, taut feeling in precordia, nausea, dizziness, and orthostatic hypotension were reduced. Therefore, combined application nitroglycerin could compensate the Western medicine, improve the clinical efficiency, and reduce the adverse events. Results of the present study provide for future clinical application of ginkgo-damole and nitroglycerin.

In addition, some limitations of this article should be noted. (1) Of all the tests included in the present meta-analysis, three experiments did not mention randomised grouping [11, 16, 23]. Although the remaining studies did so, the majority did describe the particular methods for randomisation, which may lead to self-selection bias. (2) Studies included in the present meta-analysis did not mention the preassessment method of sample size. Therefore, it is unclear whether the sample size satisfies the requirement for clinical studies. (3) Included studies did not mention allocation concealment and blinding method nor did they present report of treatment intention analysis and quit. (4) Well-designed studies assessing the curative effect of combined ginkgo-damole and nitroglycerin on hypertensive cerebropathy were not found, thus influencing poor quality of the publications included in the present meta-analysisnitroglycerin. (5) Included literature lacks detailed records of cases withdrawing from the trial. (6) The high heterogeneity of DSB and SBP data analysis may also affect the reliability of results. Furthermore, even if our search strategy is systematic and rigorous, some studies may have been missed due to language or other reasons.

## 5. Conclusions

In summary, combined administration of ginkgo-damole and nitroglycerin led to similar diastolic pressure reduction as sodium nitroprusside, while significantly reducing systolic pressure and enhancing clinical efficacy with mild adverse responses. It also has a comparable clinical safety to sodium nitroprusside. Our results support the use of combined ginkgo-damole and nitroglycerin for hypertensive cerebropathy. However, due to poor quality of the RCTs included, much stricter RCTs are needed to reduce the heterogeneity of results and avoid publication bias.

## Figures and Tables

**Table 1:** Characteristics of included articles.

Study	Year	Treatment	Control	Number (T/C)	Results report
Pu Shanji	2017	Routine treatment + nitroglycerin (ivgtt, qd) + ginkgo-damole (20 mL/d, ivgtt, qd)	Routine treatment + sodium nitroprusside (ivgtt, qd) + valsartan (80 mg, qd, po)	43/43	DBP, SBP, clinical efficacy, safety

Li Zhenfei	2017	Nitroglycerin (ivgtt, qd) + ginkgo-damole (20 mL/d, ivgtt, qd) + oral antihypertensive drugs	Sodium nitroprusside (ivgtt, qd) + oral antihypertensive drugs	60/60	DBP, SBP, clinical efficacy

Tan Shifeng	2015	Nitroglycerin (ivgtt, qd) + ginkgo-damole (20 mL/d, ivgtt, qd)	Sodium nitroprusside (ivgtt, qd) + oral antihypertensive drugs	40/40	DBP, SBP, clinical efficacy

Hu Shuhua	2014	Routine treatment + nitroglycerin (ivgtt, qd) + ginkgo-damole (20 mL/d, ivgtt, qd)	Routine treatment + sodium nitroprusside (ivgtt, qd) + valsartan (80 mg, qd, po)	49/49	DBP, SBP, clinical efficacy, safety

Li Jigui	2014	Nitroglycerin (ivgtt, qd) + ginkgo-damole (20 mL/d, ivgtt, qd) + oral antihypertensive drugs	Sodium nitroprusside (ivgtt, qd) + oral antihypertensive drugs	48/48	DBP, SBP, clinical efficacy, safety

Yuan Ge	2008	Nitroglycerin (ivgtt, qd) + ginkgo-damole (20 mL/d, ivgtt, qd) + oral antihypertensive drugs	Routine treatment + sodium nitroprusside (ivgtt, qd) + oral antihypertensive drugs	32/29	DBP, SBP, clinical efficacy, safety

SUN Yinghang	2017	Nitroglycerin (ivgtt, qd) + ginkgo-damole (20 mL/d, ivgtt, qd) + oral antihypertensive drugs	Sodium nitroprusside (ivgtt, qd) + oral antihypertensive drugs	36/36	DBP, SBP, clinical efficacy, safety

Jin Hua	2015	Routine treatment + nitroglycerin (ivgtt, qd) + ginkgo-damole (20 mL/d, ivgtt, qd)	Routine treatment + sodium nitroprusside (ivgtt, qd) + valsartan (80 mg, qd, po)	70/70	DBP, SBP, clinical efficacy

Sun Xuegong	2016	Nitroglycerin (ivgtt, qd) + ginkgo-damole (20 mL/d, ivgtt, qd) + oral antihypertensive drugs	Sodium nitroprusside (ivgtt, qd) + oral antihypertensive drugs	46/46	DBP, SBP, clinical efficacy

Chen Xishan	2011	Nitroglycerin (ivgtt, qd) + ginkgo-damole (20 mL/d, ivgtt, qd) + oral antihypertensive drugs	Sodium nitroprusside (ivgtt, qd) + oral antihypertensive drugs	30/30	DBP, SBP, clinical efficacy

Zhu Feijuan	2017	Nitroglycerin (ivgtt, qd) + ginkgo-damole (20 mL/d, ivgtt, qd)	Sodium nitroprusside (ivgtt, qd) + oral antihypertensive drugs	40/40	DBP, SBP, clinical efficacy, safety

Wang Junhong	2016	Routine treatment + nitroglycerin (ivgtt, qd) + ginkgo-damole (20 mL/d, ivgtt, qd)	Routine treatment + sodium nitroprusside (ivgtt, qd) + oral antihypertensive drugs	45/45	Clinical efficacy

Min Riuxue	2015	Mannitol 125–250 ml + nitroglycerin (5 mg/d, ivgtt, qd) + ginkgo-damole (20 mL/d, ivgtt, qd) + oral antihypertensive drugs	Mannitol 125–250 ml + sodium nitroprusside (50 mg/d, ivgtt, qd) + oral antihypertensive drugs	31/31	Clinical efficacy

Yu Qisheng	2014	Nitroglycerin (ivgtt, qd) + ginkgo-damole (20 mL/d, ivgtt, qd)	Sodium nitroprusside (ivgtt, qd) + oral antihypertensive drugs	40/40	Clinical efficacy

Xin Zhaoyang	2013	Nitroglycerin (ivgtt, qd) + ginkgo-damole (20 mL/d, ivgtt, qd) + oral antihypertensive drugs	Sodium nitroprusside (ivgtt, qd) + oral antihypertensive drugs	90/90	Clinical efficacy

Zhang Mingchao	2016	Routine treatment + nitroglycerin (5 mg/d, ivgtt, qd) + ginkgo-damole (20 mL/d, ivgtt, qd) + oral antihypertensive drugs	Routine treatment + sodium nitroprusside (ivgtt, qd) + oral antihypertensive drugs	55/55	Clinical efficacy, safety

## Data Availability

The data supporting this meta-analysis are from the previously reported studies and datasets, which have been cited. The data used to support the findings of this study are available from the corresponding author upon request.
